# Provision of pest alerts is associated with better farm performance in Africa

**DOI:** 10.1002/ps.70196

**Published:** 2025-09-06

**Authors:** Makaiko G Khonje, Justice A Tambo, Bryony Taylor, Charlotte Day, Frances Williams

**Affiliations:** ^1^ CABI Nairobi Kenya; ^2^ CABI Delémont Switzerland; ^3^ CABI Wallingford UK

**Keywords:** pest risk information, farm performance, cross‐sectional data, Africa

## Abstract

**BACKGROUND:**

Crop pests cause substantial crop yield and economic losses, food insecurity, and negative impacts on human health and environment globally. Timely provision of pest risk alerts – that is, the optimum time to intervene against key pests before invasion or establishment – to smallholder farmers on pest management could improve farm performance. However, there is little quantitative evidence testing this hypothesis.

**RESULTS:**

To address this gap, we use primary survey data from over 4000 smallholder farmers across four African countries: Ghana, Kenya, Malawi, and Zambia. Our results suggest that providing pest alerts to smallholder farmers is associated with a higher probability of adopting integrated pest management (IPM) practices by 8–32 percentage points, as well as an increase in crop yield and income by 18–26%.

**CONCLUSION:**

Improving timely access to information on pest risks and management could have substantial benefits on farm productivity and income in Africa. Our findings provide a practical pathway on how pest‐induced crop losses could be minimized by addressing informational barriers. © 2025 The Author(s). *Pest Management Science* published by John Wiley & Sons Ltd on behalf of Society of Chemical Industry.

## Graphical abstract

Provision of pest alerts on invasive crop pest control in Africa was linked with a higher likelihood of adopting integrated pest management practices, as well as an improvement in crop yield and income. The results suggest that pest alerts are crucial in sustainable pest management strategies against invasive pests.

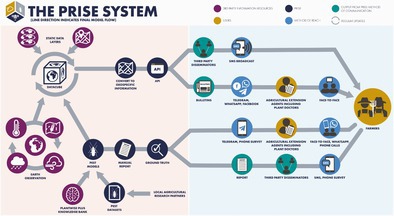

## 1 INTRODUCTION

Crop pests remain an outstanding agricultural problem worldwide.^1^, ^2^ Up to 40% of global crops are lost to pests, diseases, and weeds, with invasive species causing a global economic loss of about US$ 1.3 trillion over the past five decades.^3^, ^4^ In Africa, invasive crop pests and diseases are associated with annual economic losses of about US$ 66 billion,^5^ with the largest contributors being fall armyworm (FAW, *Spodoptera frugiperda*) and tomato leaf miner (TLM, *Phthorimaea absoluta*).^5^, ^6^, ^7^, ^8^ In addition, pests are associated with increased food insecurity, and negative impacts on human health and environment through pesticide use intensification.^3^, ^4^, ^6^, ^7^, ^8^, ^9^ Unfortunately, these economic losses and externalities are likely to increase because of the predicted increase in impact of pests due to climate change, trade globalization, agricultural intensification, and poor access to effective and control methods.^1^, ^10^, ^11^

Timely provision of agricultural information on crop pests and management can prevent pest populations from building up and minimizing crop yield, economic and livelihood losses.^5^, ^11^ For example, a few pilot projects aimed at providing pest alerts – defined as the optimum time‐to‐act on crop pests before pest invasion or establishment – to smallholder farmers have been implemented in four Africa countries: Ghana, Kenya, Malawi, and Zambia.^12^, ^13^ Here, through an initiative known as the Pest Risk Information Services (PRISE), site‐specific pest advisories were disseminated to smallholder farmers via multiple information channels such as short message services (SMS), radio, community information centers (CICs), and plant clinics. To generate pest alerts, this initiative uses a novel combination of earth observations, weather satellite data, plant–pest lifecycle, and real‐time field observations. Overall, emerging evidence suggests that the provision of pest alerts to smallholder farmers stimulated the adoption of integrated pest management (IPM) practices and improved crop yields and incomes.^12^, ^13^

Even though literature examining the linkage between pest information and farm performance is growing, existing studies have certain limitations. Only a handful of studies^9^, ^14^, ^15^, ^16^, ^17^ have analyzed the linkage between pest information and farm performance, but none of these studies focused on pest (PRISE) alerts. We are aware of only one study^13^ that also analyzed the relationship between pest alerts and maize productivity in Ghana. They found that pest alerts via CICs had positive associations with IPM and maize yield and income. However, most of these prior studies^9^, ^13^, ^14^, ^15^, ^16^, ^17^ use data from individual countries or specific locations within these countries, so broader conclusions – that is, limited external validity – beyond these specific settings are hardly possible. Moreover, the pest alert interventions in Tambo *et al*.^13^ relied on CICs to broadcast alerts, while the current study was based on multiple information delivery methods.

In the present study, we address these caveats and extend the scope of the previous studies by analyzing the relationship between pest alerts and farm performance – measured by adoption of IPM, crop yield, and crop income – using cross‐sectional data from four African countries: Ghana, Kenya, Malawi, and Zambia. A focus on Africa is important, not only because of high prevalence rate for food insecurity and poverty, but also because of recent high reported losses from invasive crop pests as well as those experienced from indigenous pests.^5^, ^6^, ^7^, ^8^, ^18^, ^19^, ^20^ For example, pests were associated with reduction in crop yields and income by 22–72% in several sub‐Saharan African (SSA) countries including Ghana, Ethiopia, Kenya, Rwanda, Tanzania, Uganda, Malawi, and Zambia.^5^, ^6^, ^7^, ^8^, ^18^, ^19^, ^20^ Moreover, due to poor digital infrastructure, timely access to information on pest risks and management is difficult in many rural parts of Africa.^13^, ^14^, ^18^, ^21^, ^22^

## 2 MATERIALS AND METHODS

### 2.1 Contextual background

To minimize pest‐induced crop losses, CABI in collaboration with Assimila Ltd and several international partners, initiated the PRISE project. PRISE was a 5‐year project (2017–2022), funded by the UK Space Agency's International Partnerships Program. As earlier mentioned, the project was developed and implemented in four SSA countries: Ghana, Kenya, Malawi, and Zambia. In each country, pest alerts on key crop pests along with best practice management advice were disseminated to smallholder farmers and other agricultural stakeholders in a specific community during the 2020/2021 growing season.

The project worked with several local partners in each country. For example, for Ghana, the initiative worked with Plant Protection and Regulatory Services Directorate of the Ministry of Agriculture (MoA). In Kenya, the project worked with three local partners: the Kenya Agriculture and Livestock Research Organization, the Kenya Meteorological Department and the MoA. In Malawi, the initiative worked with the Department of Agricultural Research Services under the MoA as well as other local institutions such as Farm Radio Trust. While in Zambia, the project worked with a government‐owned institution known as the Zambia Integrated Agriculture Management Information System as a local partner.

Providing pest alerts to smallholder farmers on when acting against a pest attack is going to be most effective is important in several ways. (i) This could increase farmer's resilience to crop losses caused by pest attacks, and (ii) it could also increase efficacy of intervention measures; that is, moving away from curative to preventative control approaches. (iii) Reducing detrimental effects of crop pests – before the irreversible damage is caused – may also help farmers to realize higher yields and contribute to achieve multiple sustainable development goals such as no poverty and zero hunger.

Although several destructive crop pests exist,^5^, ^6^, ^7^, ^8^ the PRISE project largely focused on three crop pests: FAW, bean fly (*Ophiomyia phaseoli*), and TLM. Relatedly, this study targeted three crops: maize, beans, and tomato for several reasons. First, they are commonly produced together in a mixed‐maize cropping system across multiple African countries.^6^ Second, these crops were selected as priority crops by national stakeholders in the implementing countries. Third, emerging evidence suggest that pest infestations for these crops are associated with higher yield losses.^5^, ^6^, ^7^, ^8^, ^18^, ^19^, ^20^ While FAW was a target pest in all the four study countries, the other two crop pests were only targeted in Kenya, Malawi, and Zambia (Supporting Information Table S1).

### 2.2 Study area (region)

In all the four study countries, household surveys were conducted where the PRISE interventions were implemented. Precisely, in Ghana, the study was conducted in the following areas: Berekum Municipal, Dormaa East, Kintampo South, Nkoranza North, Techiman North, Wenchi, Sunyani Municipal, Sunyani West, Tain, and Nkoranza South. While in Kenya, the study was done in five counties or districts namely Bungoma, Embu, Kirinyaga, Murang'a and Tharaka‐Nithi. For Malawi, the study was implemented in five districts: Mzimba, Dedza, Nkhotakota, Balaka, and Mulanje. Lastly, in Zambia, the study was conducted in the following districts: Chongwe, Luangwa, Katete, Petauke, Limulunga, Nkeyema, Mansa, Samfya, Mbala, Mpulungu, Mpika, Nakonde, Mumbwa, Shibuyunji, Gwembe, and Mazabuka.

### 2.3 Sampling strategy and sample size

To select farming households for the interviews during the survey, a multi‐stage sampling technique (involving three stages) in each of the study countries was used. In the first stage, focusing on key producing districts for the three target crops, we randomly sampled districts or counties from the list of districts or counties where the PRISE project was implemented.

In the second stage, a cluster sampling approach to select enumeration areas and villages was used. The sampled enumeration areas – also known as communities in Ghana or extension planning areas in Malawi or wards in Kenya or camps in Zambia – were then grouped into two clusters: (i) PRISE intervention villages (e.g., where farmers received information on good agricultural practices and time‐to‐act messages on a particular pest from the PRISE project) and (ii) non‐PRISE intervention villages. However, to minimize the risk of information spillover between treatment and control farmers, we ensured that the selected intervention (PRISE) villages were not geographically adjacent to non‐intervention villages.

In the third stage, farmers were randomly sampled from each of the cluster villages using probability proportional to size sampling procedure. That is, the final sample size for each enumeration area was proportional to the size of its population. Although randomization was done at the village level, we also used weighted estimators as suggested by Solon *et al*.^23^ to account for potential selection bias. After accounting for outliers, the final sample size (number of households) for each country in this study include 880 (Ghana), 1413 (Kenya), 819 (Malawi), and 942 (Zambia). A detailed sample distribution is shown in Table S2.

### 2.4 Survey questionnaire

To make our analyses comparable and consistent, the study used a similar questionnaire across study countries. Household‐level information on demographic structure, asset ownership especially, information and communication technology (ICT)‐based assets, IPM practices, crop yields, economic (farm income) activities, and other features were collected using well‐trained supervisors and enumerators. To further improve data quality, the study used computer‐aided personal interviews. Key to this study, we also collected data on farmer's access to the PRISE – ‘pest’ – alerts.

### 2.5 Measuring pest alerts

In this study, our main explanatory (treatment) variable of interest is access to pest alerts by smallholder farmers, which is measured as a binary variable. This variable takes a value of 1 if a farmer received pest (PRISE) alert message, and 0 otherwise. Here, all sampled farmers from the treatment (PRISE) villages received information on good agricultural practices and optimum time to act against a particular pest. As previously mentioned, due to variability in digital infrastructure, the pest alerts were disseminated through various information channels including text or voice SMSs, community radios, CICs, and plant clinics in each country (see Table S1). For brevity, a detailed variable description for other explanatory (independent) variables of interest is presented in Section 3.1 .

**Table 1:** Descriptive statistics by country

	Ghana	Kenya	Malawi	Zambia
	(1)	(2)	(3)	(4)
*Farm performance (dependent variables)*				
Use of IPM practices (1,0)	0.68	0.66	0.44	0.18
	(0.47)	(0.47)	(0.50)	(0.39)
Use of IPM practices (count)	3.12	3.18	2.78	2.29
	(0.96)	(0.98)	(1.05)	(0.74)
Maize yield (kg/ha)	1447	2409	1383	2420
	(1094)	(1825)	(1117)	(2492)
Crop income (US$/ha)	721	612	113	196
	(985)	(540)	(194)	(1088)
Net crop income (US$/ha)	509	515		89
	(521)	(540)		(1121)
*Explanatory (independent) variables*				
HH received pest alert message (1,0)	0.41	0.62	0.35	0.43
	(0.50)	(0.49)	(0.48)	(0.50)
HH owned an ICT‐based asset (1,0)	0.95	0.69	0.14	0.80
	(0.21)	(0.46)	(0.35)	(0.41)
Total farm size (acres)	5.49	1.50	2.76	6.48
	(3.15)	(1.18)	(2.70)	(11.43)
Observations (number of households)	880	1413	819	942

Crop income is generated from the three target crops: maize, beans, and tomato. Mean values are shown with standard deviations in parentheses. The exchange rates for a US$ to local currencies at the time of the survey were 6.24, 110, 824 and 10 for Ghana, Kenya, Malawi, and Zambia, respectively. Full weighted descriptive results and unweighted descriptive results are shown in Table S3.

Abbreviations: IPM, integrated pest management; ICT, information and communication technology; HH, household head.

### 2.6 Measuring farm performance

To measure farm performance, the study uses three indicators as dependent variables. First, a count and binary variable on use of IPM practices is generated in order to measure both intensive and extensive margins, respectively. Specifically, IPM is measured as the count of five broad practices: (i) chemical, (ii) mechanical, (iii) cultural, (iv) biological control using predators, and (v) biopesticides adopted by farmers during a cropping season. In each study country, the questionnaire had a unified module (a series of structured questions) on IPM practices. Overall, the maximum number of common practices in each of the five IPM components were two, resulting in ten adopted practices. Based on these major practices, we then calculated a binary IPM variable taking a value of 1 if a farming household had adopted at least one of the five IPM practices, and 0 otherwise. The use of these five components is widely used to measure adoption of IPM practices in the SSA.^8^, ^9^, ^13^, ^24^, ^25^, ^26^

Second, yield calculations were performed for the three study crops namely maize, beans, and tomato. For each one of the crops, crop yield (kg/ha) was measured as a ratio of total harvest in kilograms to farm size under the crop in hectares. However, the survey questionnaire for Ghana captured detailed information on maize only.

Third, the study used crop income (value of crop production) as a proxy for farm performance. Here, crop income (US$/ha) for the three target crops (as opposed to a single crop only) planted by the farming household is calculated as the ratio of total crop harvest – price (US$/kg) multiplied with quantity produced (kg) – to unit area (ha). Price data were also collected during the household surveys in all the four study countries. To account for production costs (i.e., seed, fertilizer, and pesticides) in our income calculations, net crop income – gross income minus production costs – was calculated for Ghana, Kenya, and Zambia. However, this could not be done for Malawi as expenditure data on major inputs were not captured.

Despite using crop yield as a better proxy measure for productivity, we also focused on the use of crop income (USS/ha) for two reasons. Not only did it help us to deal with additive challenges associated with crop yields across target crops, but also more often farmers grow multiple crops or even adopt inter‐cropping on the same piece of land. Thus, this feature makes it difficult to attribute returns to land or labor to individual crops only.^27^, ^28^

Nevertheless, this indicator has some caveats. First, farmers may forget the true cost of input as they rarely keep farm records.^27^, ^29^ Second, adding input expenditure data requires some level of literacy and numerical skills. Yet, most farmers lack these skills in rural parts of SSA.^30^, ^31^ Third, crop income (USS/ha) varies across countries because of (i) availability of arable land relative to population size,^32^, ^33^, ^34^, ^35^ (ii) climate change and other production shocks,^36^ (iii) infrastructure, (iv) economic shocks and price volatility, and (v) market distortions (subsidies).^27^

### 2.7 Statistical methods

We estimate associations between pest alerts and farm performance with cross‐sectional data of the following type:

(1) $${\mathrm{FP}}_{\mathrm{hj}}=\alpha +\beta {\text{PRISE}}_{\mathrm{hj}}+\gamma X_{\mathrm{hj}}+\varepsilon_{\mathrm{hj}}$$

where ${\mathrm{FP}}_{\mathrm{hj}}$ is farm performance indicator – measured by adoption of IPM, crop yield, and crop income – of household *h* in country *j*. ${\text{PRISE}}_{\mathrm{hj}}$ is access to pest alerts for household *h* in country *j*, measured as a binary variable as described in Section 2.5. The parameter of particular interest is β. A positive value for β implies that provision of pest alerts to farmers is associated with increased farm performance, which is also our hypothesis to be tested in this study.

To estimate Eqn (1), several household characteristics ($X_{\mathrm{hj}}$) were included as control (independent) variables. Specifically, the following variables were included: ownership of ICT‐based assets including phones, radio, and television, total landholding size, gender, age, and education of the household head, weather shocks, and institutional factors, including access to credit and subsidized inputs. In addition, the term $\varepsilon_{\mathrm{hj}}$ is a random error term clustered at the enumeration area to account for the remaining institutional heterogeneity and other village‐specific characteristics.^37^ Despite including several controls in Eqn (1), other sources of endogeneity may still exist. Thus, we interpret β as mere associations.

More broadly, to estimate Eqn (1), three different regression models are used, depending on attributes of the dependent variable. First, for dependent variables that are measured with count data (i.e., number of IPM practices), Poisson estimators are used.^38^ Second, for dependent variables that are binary, probit or linear probability model (LPM) estimators are used. Third, for dependent variables that are continuous and normally distributed including crop yields and income, ordinary least squares (OLS) estimators are used. Generally, we estimated Eqn (1) for each country for two reasons. First, the type of PRISE intervention was slightly different in each country. Second, different information channels were used to disseminate pest alerts across the study countries.

## 3 RESULTS

### 3.1 Descriptive results

Table 1 shows farm performance indicators and selected explanatory variables of interest, underlining that crop yields are still lower than the maximum potential of at least 5 t/ha especially for maize.^27^, ^35^ Similarly, crop income (US$/ha) is the lowest in Malawi compared to Ghana, which had the highest (721) average crop income. Zambia had the lowest number of farmers (18% of the sampled farming households) who had adopted IPM practices.

**Figure 1:**
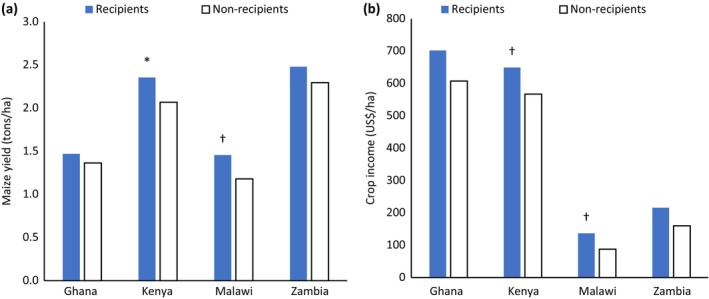
Crop productivity and income by recipient of pest alerts. (a) Maize yield. (b) Crop income. The *t*‐tests were carried out to test for mean differences between recipients and non‐recipients of pest alerts. *N* = 880, 1413, 819 and 942 for Ghana, Kenya, Malawi, and Zambia, respectively. * and † indicate statistical significance at 10% and 1% level, respectively.

On average, we also found that access to pest alerts range between 35% in Zambia and 62% in Kenya (Table 1). Higher access to pest alerts seems to be associated with higher maize yields in countries like Kenya. Statistically, this is confirmed in Fig. 1(a), where we found that farmers who received pest alerts had higher maize yield than non‐recipients in Kenya and Malawi. A similar pattern is observed for crop income in Kenya and Malawi (Fig. 1(b)). In contrast, the results for Ghana and Zambia are positive but statistically insignificant.

While these preliminary results provide early evidence on positive associations between pest alerts and farm performance, they do not account for confounding factors. To address this caveat, we next report main regression results from Eqn (1). In these models, we control household characteristics, household wealth, and other socioeconomic characteristics.

### 3.2 Pest alerts and IPM practices

Do farmers really benefit from timely access to pest alerts? To answer this research question, we report results from Eqn (1) in three ways. As an immediate outcome associated with provision of pest alerts, our quest is first to understand whether pest alerts (i) stimulated adoption of IPM practices. We then focus on whether these immediate outcomes associated with pest alerts improved (ii) crop productivity and (iii) income. Next, we present the three main results in details.

**Figure 2:**
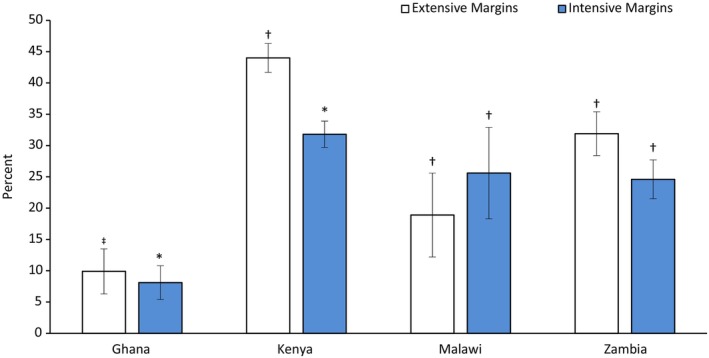
Predicted associations between pest alerts and use of integrated pest management (IPM) practices. The dependent variable in all the models is either a binary (1/0) variable or a count variable for IPM practices. Marginal effects from linear probability model (LPM) and Poisson regression models are shown with robust standard error bars clustered at enumeration area. Full model results are shown in Supporting Information Tables S4 and S5. *N* = 880, 1408, 819, and 942 for Ghana, Kenya, Malawi, and Zambia, respectively. *, ‡, and † indicate statistical significance at 10%, 5%, and 1% level, respectively.

First, Fig. 2 presents summarized regression results on the relationship between provision of pest alerts and use of IPM practices. Overall, we find that timely access to pest alerts was associated with an increase in the likelihood of adopting IPM practices, ranging from 8% points in Ghana to 32 percentage points in Kenya (Fig. 2). As a robustness check, we report both extensive (probability to use IPM practices) margins and intensive (count of IPM practices) margins in Fig. 2. In general, these results are consistent with some of the emerging evidence on the pivotal role of information campaigns in enhancing farmers' knowledge on pest risks and management, particularly during the outbreak of a new invasive crop pest.^13^, ^15^, ^16^, ^17^

Because our pest alerts promoted use of several IPM practices including spraying of biopesticides, uprooting/removing affected plants after scouting the field, conserving natural enemies, use of pheromone traps, using botanical extracts, planting repellent plants, removing debris or burning or burying affected plants, and cultural methods, this could directly reduce crop pest population or attack and crop losses. Moreover, use of non‐chemical IPM practices could enhance safer food production and reduce chemical food contamination.

One important policy implication drawn from these findings (i.e., Fig. 2) is that enhancing access to information on pest risks and management through different information channels could have substantial benefits on adoption of IPM practices in selected developing countries. Moreover, one strand of literature^9^, ^13^, ^14^, ^21^ also suggests that IPM practices are not widely adopted in low‐ and middle‐income countries, mostly due to lack of information, especially pest alerts. However, for this policy proposal to be effective, it requires addressing other structural challenges including poor digital infrastructure, high cost to access ICT services, unreliable energy sources, and other structural barriers.^9^, ^13^, ^14^, ^21^ Furthermore, the novel use of pest alerts model could be among standard models to be replicated in other developing countries, which are also prone to invasive pest attacks.

### 3.3 Pest alerts and crop productivity

Second, we attempt to address this research question: Can the dissemination of pest alerts be associated with increased farm productivity? Table 2 presents summarized regression results in which, maize yield, bean yield and tomato yield, and crop income, respectively, are the dependent variables, and farmers' access to pest alerts is our main explanatory variable of interest.

**Table 2:** Predicted associations between pest alerts and crop yields: ordinary least squares (OLS) results

Dependent variable	Maize yield (IHS)	Bean yield (IHS)	Tomato yield (IHS)
Country	(1)	(2)	(3)
Ghana (*n* = 876)	0.230***		
	(0.068)		
Kenya (*n* = 885)	0.197***	0.237***	
	(0.055)	(0.084)	
Malawi (*n* = 812)	0.194**	0.243**	0.433*
	(0.078)	(0.115)	(0.220)
Zambia (*n* = 940)	0.064	0.091	0.329
	(0.055)	(0.142)	(0.384)

Coefficient estimates from OLS regression models on the effect of pest alerts (1/0) are shown with robust standard errors clustered at an enumeration area in parentheses. Separate models were estimated for each country and for each of the three dependent variables. The dependent variables were transformed using inverse an inverse hyperbolic sine (IHS) transformation, that is ($\log\left(x+{\left(x^{2}+1\right)}^{0.5}\right)$). Full model results with relevant confounding factors are shown in Supporting Information Tables S6 and S7.

* *P* < 0.1.

** *P* < 0.05.

*** *P* < 0.01.

A few important insights emerge from Table 2. We found that access to pest alerts is positively and significantly associated with an increase in maize yield by roughly 23%, 20%, and 19% in Ghana, Kenya, and Malawi, respectively, which is consistent with our descriptive results in Fig. 1(a) as previously explained. For other crop yield model results, we found that access to pest alerts was associated with an increase in bean yields and tomato yields only in Kenya and Malawi by approximately 24‐43% (Table 2). The results for Zambia are positive but statistically insignificant. In summary, these results are in line with experimental evidence from Africa,^39^, ^40^, ^41^, ^42^ where it was found that ICT‐based extension approaches increased crop (i.e., maize, rice and legume) yield via adoption of fertilizer management practices,^43^ ranging from 7% to 24%. However, these studies did not focus on pest alerts as we do.

### 3.4 Pest alerts and crop income

**Figure 3:**
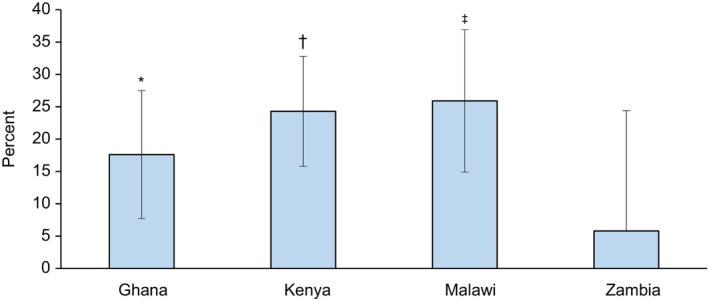
Predicted associations between pest alerts and crop income. Marginal effects from ordinary least squares (OLS) are shown with robust standard error bars clustered at enumeration area. Full model results are shown in Supporting Information Table S8. *N* = 880, 942, 819 and 942 for Ghana, Kenya, Malawi, and Zambia, respectively. *, ‡, and † indicate statistical significance at 10%, 5%, and 1% level, respectively.

Our third and last important result analyzes the relationship between pest alerts and crop income. The results are illustrated in Fig. 3. Consistent with crop yield model results and a study done by Tambo *et al*.,^13^ we also found that provision of pest alerts to smallholder farmers is associated with an increase in crop income in Ghana, Kenya, and Malawi by 18–26% (Fig. 3). Crop income results for Zambia are still positive, but they are not statistically significant. We also get similar results for net crop income for Ghana, which are reported in Table S9.

### 3.5 Robustness checks

Several alternative models were estimated as robustness checks. First, to check the consistency of Fig. 2 results, we used a standard probit model instead of LPM estimators. The results are reported in Table S10. We also found that pest alerts is associated with an increase in the likelihood of adopting IPM practices by at least 33 percentage points.

Second, to account for potential endogenous sampling and correcting for heteroskedasticity, we used weighted estimators as suggested by Solon *et al*.^23^ We used a ratio of district population to the total population of the sampled districts as sample weights. The results are reported in Tables S11 and S12 for IPM practices, and crop productivity and income, respectively.

Lastly, we also estimated a treatment effect model: an inverse probability weighted regression adjustment (IPWRA), and the results are shown in Table S13. Generally, these results aligns with our main results (Table 2, Figs 2 and 3), suggesting that providing pest alerts to smallholder farmers was associated with an increase in the likelihood of adopting IPM practices by 9–42 percentage points, as well as an increase in crop yield and income by roughly 19–26%.

## 4 DISCUSSION AND CONCLUSION

With the digital revolution, providing timely information on pest risk and management to smallholder farmers through various information channels could have substantial benefits on farm productivity and income. However, providing quantitative evidence on this claim in a multi‐country setting is still scanty. Our study adds to the existing literature by analyzing the relationship between provision of pest alerts to smallholder farmers and farm performance using primary survey data from four African countries: Ghana, Kenya, Malawi, and Zambia. Specifically, we have addressed two research questions: (i) Does the provision of pest alerts to smallholder farmers enhance adoption of IPM practices? (ii) Can the dissemination of pest alerts be associated with increased crop productivity and income?

Our results suggest that providing pest alerts to smallholder farmers via various information channels is significantly and positively associated with farm performance outcomes. After accounting for other confounding factors, we have shown that timely access to pest alerts is associated with the higher likelihood of adopting IPM practices, ranging from 8 to 32 percentage points. Even though there is limited multi‐country evidence on predicted effects of pest alerts, our findings are consistent with general literature^16^, ^17^, ^21^, ^43^ on pest information interventions.

Furthermore, we find evidence that timely access to pest alerts was associated with an increase in crop yield and crop income in Ghana, Kenya, and Malawi, ranging from 18% to 26%. A plausible mechanism is that provision of pest alerts enhanced timely management of crop pests, which ultimately increased crop yields and income. Not only are these findings reasonable but also unique and important. Previous studies have shown that crop pest outbreaks are associated with crop yield and income losses, ranging from by 22% to 72% in SSA.^5^, ^6^, ^7^, ^8^, ^18^, ^19^, ^20^ Because our interventions targeted management of different crop pests, we can speculate that provision of pest alerts – due to poor digital infrastructure, this is a major constrain for most farmers in the Global South^8^, ^14^, ^17^, ^18^, ^19^, ^22^, ^43^, ^44^, ^45^, ^46^ – to smallholder farmers is among potential solutions for crop pest attacks and its losses. Therefore, one crucial policy implication drawn from our results is that improving farmers' access to information on pest risks and management could have substantial benefits on use of IPM practices, crop productivity, and income in selected developing countries.

Beyond this general lesson, our study also has some caveats. First, our empirical analysis is not causal; we did not use experimental evaluation methods and panel data, which is ideal to account for unobserved heterogeneity. Second, even though our selected intervention (PRISE) villages were not geographically adjacent to non‐intervention villages, there is a possibility of information spillovers. This may lead to an underestimation of the predicted relationships between pest alerts and farm performance outcomes. Therefore, it would be useful for future research to use experimental designs that properly account for potential spillover effects. Third, although comparable survey tools were used in the four study countries, the actual survey methodologies were slightly different. Specifically, while we used in‐person interviews in Ghana, Malawi and Zambia, telephone interviews were used for Kenya due to COVID‐19 restrictions. This limited the ability for enumerators to physically observe and validate yield data. Fourth, due to data limitation, the study was unable to determine potential non‐users of pest alerts within the treatment enumeration area. Relatedly, it is well known that crop yield and income are prone to measurement errors as most farmers use recall data and non‐standard units.^28^, ^29^, ^30^, ^33^, ^34^, ^47^ Moreover, availability of arable land relative to population size,^32^, ^33^, ^34^ input subsidies,^27^ climate change,^36^ infrastructure, and economic shocks including volatility in commodity prices varies in the four study countries. Hence, with these factors, we might have underestimated or overestimated crop yield and income. Finally, due to variability in digital infrastructure across the four study countries, we used different information channels to disseminate pest alerts. As a result, we could not run a unified regression model for all four study countries. Therefore, addressing these caveats could be a useful foundation for future research.

## AUTHOR CONTRIBUTIONS

MGK, JAT, and FW conceptualized and designed the study. MGK curated the data and carried out the data analysis, with input from JAT and FW. MGK visualized the results and wrote the manuscript. All authors reviewed, edited and provided comments on the manuscript.

## CONFLICT OF INTEREST

The authors declare no competing interests.

## Supporting information


**Table S1.** Target pests and typology of information channels for pest alerts.
**Table S2.** Number of sampled households by country, region and district or county.
**Table S3.** Descriptive statistics of key variables in the analysis.
**Table S4.** Predicted associations between pest alerts and use of IPM practices (extensive margins).
**Table S5.** Predicted associations between pest alerts and use of IPM practices (intensive margins).
**Table S6.** Predicted associations between pest alerts and maize yield.
**Table S7.** Predicted associations between pest alerts and bean yield and tomato yield.
**Table S8.** Predicted associations between pest alerts and crop income.
**Table S9.** Predicted associations between pest alerts and net crop income.
**Table S10.** Predicted associations between pest alerts and use of IPM practices: summarized probit models.
**Table S11.** Predicted associations between pest alerts and use of IPM practices: summarized weighted models.
**Table S12.** Predicted associations between pest alerts and crop productivity and income: summarized weighted regression model results.
**Table S13.** Predicted associations between pest alerts and use of IPM practices, maize yield, and income: IPWRA models.

## Data Availability

The data that support the findings of this study are available from the corresponding author upon reasonable request.
