# Supplementary material for: Provision of pest alerts is associated with better farm performance in Africa
Source: Pest Manag Sci. 2025 Sep 6;82(1):340–7. doi: 10.1002/ps.70196 (PMC12713714; doi:10.1002/ps.70196)
Supplement: Supplementary file 1 — Table S1. Target pests and typology of information channels for pest alerts. Table S2. Number of sampled households by country, region and district or county. Table S3. Descriptive statistics of key variables in the analysis. Table S4. Predicted associations between pest alerts and use of IPM practices (extensive margins). Table S5. Predicted associations between pest alerts and use of IPM practices (intensive margins). Table S6. Predicted associations between pest alerts and maize yield. Table S7. Predicted associations between pest alerts and bean yield and tomato yield. Table S8. Predicted associations between pest alerts and crop income. Table S9. Predicted associations between pest alerts and net crop income. Table S10. Predicted associations between pest alerts and use of IPM practices: summarized probit models. Table S11. Predicted associations between pest alerts and use of IPM practices: summarized weighted models. Table S12. Predicted associations between pest alerts and crop productivity and income: summarized weighted regression model results. Table S13. Predicted associations between pest alerts and use of IPM practices, maize yield, and income: IPWRA models. [file PS-82-340-s001.pdf]

Provision of pest alerts is associated with better farm performance in Africa

Makaiko G. Khonje*, Justice A. Tambo, Bryony Taylor, Charlotte Day & Frances Williams

*Corresponding author. Tel.: +254 113 731 960/+265 999 750 940/+265 888 620 239;

Email: m.khonje@cabi.org/makakhonje@gmail.com

Supplementary Information

Contents

Supplementary Table S1 | Target pests and typology of information channels for pest alerts 1

Supplementary Table S2 | Number of sampled households by country, region and district or county.................. 1

Supplementary Table S3 | Descriptive statistics of key variables in the analysis 2

Supplementary Table S4 | Predicted associations between pest alerts and use of IPM practices (Extensive Margins) 3

Supplementary Table S5 | Predicted associations between pest alerts and use of IPM practices (Intensive Margins) 3

Supplementary Table S6 | Predicted associations between pest alerts and maize yield... 4

Supplementary Table S7 | Predicted associations between pest alerts and bean yield and tomato yield................ 4

Supplementary Table S8 | Predicted associations between pest alerts and crop income 5

Supplementary Table S9 | Predicted associations between pest alerts and net crop income 5

Supplementary Table S10 | Predicted associations between pest alerts and use of IPM practices: Summarized Probit Models 6

Supplementary Table S11 | Predicted associations between pest alerts and use of IPM practices: Summarized Weighted Models.. 6

Supplementary Table S12 | Predicted associations between pest alerts and crop productivity and income: Summarized Weighted Regression Model Results 6

Supplementary Table S13 | Predicted associations between pest alerts and use of IPM practices, maize yield, and income: IPWRA models 6

Supplementary Table S1 | Target pests and typology of information channels for pest alerts

	Ghana	Kenya	Malawi	Zambia
	(1)	(2)	(3)	(4)
Target pest				
Fall armyworm (FAW)	Yes	Yes	Yes	Yes
Bean fly	No	Yes	Yes	Yes
Tomato leaf miner	No	Yes	Yes	Yes
Information channel				
Text SMS	No	Yes	Yes	Yes
Voice SMS	Yes	No	No	No
Radio	No	No	Yes	No
Hotlines	No	No	Yes	No
Community information centers (CICs)	Yes	No	No	No
Plant clinics (doctors)	No	Yes	Yes	Yes
Service provider	Esoko and CICs	PxD with	Farm	ZIAMIS

Note: SMS, PxD, MoA-INFO, and ZIAMIS denotes, Short message service, Precision development, Ministry of Agriculture SMS platform, and Zambia Integrated Agriculture Management Information System.

Supplementary Table S2 | Number of sampled households by country, region and district or county

Country	Region/Province	District/County	Number of sampled households
Ghana	Bono	Berekum Municipal	55
		Dormaa East	134
		Kintampo South	154
		Nkoranza North	54
		Techiman North	101
		Wenchi	99
	Bono East	Sunyani Municipal	82
		Sunyani West	72
		Tain	75
		Nkoranza South	54
		**Total**	**880**
Kenya		Bungoma	729
		Embu	321
		Kirinyaga	224
		Murang'a	62
		Tharaka-Nithi	77
		**Total**	**1,413**
Malawi	Northern	Mzimba	160
	Central	Dedza	168
		Nkhotakota	162
	Southern	Balaka	163
		Mulanje	166
		**Total**	**819**
Zambia	Lusaka	Chongwe	39
		Luangwa	42
	Eastern	Katete	90
		Petauke	92
	Western	Limulunga	41
		Nkeyema	44
	Luapula	Mansa	62
		Samfya	70
	Northern	Mbala	74
		Mpulungu	63
	Muchinga	Mpika	70
		Nakonde	69
	Central	Mumbwa	61
		Shibuyunji	65
	Southern	Gwembe	30
		Mazabuka	30
		**Total**	**942**

Supplementary Table S3 | Descriptive statistics of key variables in the analysis

Country	Ghana		Kenya		Malawi		Zambia	
	Unweighted	Weighted	Unweighted	Weighted	Unweighted	Weighted	Unweighted	Weighted
Farm performance (dependent variables)								
Use of IPM practices (1,0)	0.73	0.68	0.67	0.66	0.46	0.44	0.15	0.18
	(0.45)	(0.47)	(0.47)	(0.47)	(0.50)	(0.50)	(0.36)	(0.39)
Use of IPM practices (count)	3.20	3.12	3.25	3.18	2.82	2.78	2.24	2.29
	(0.96)	(0.96)	(0.99)	(0.98)	(1.07)	(1.05)	(0.65)	(0.74)
Maize yield (kg/ha)	1409.46	1447.39	2292.26	2408.94	1283.20	1382.66	2360.29	2419.84
	(1047.13)	(1093.86)	(1834.00)	(1824.74)	(1074.28)	(1117.01)	(2406.65)	(2491.73)
Bean yield (kg/ha)			632.97	570.88	147.96	148.44	1060.54	1061.84
			(951.16)	(801.59)	(6459.43)	(125.02)	(2652.69)	(1077.46)
Crop income (US$/ha)	647.74	720.82	616.96	612.17	105.99	112.82	179.83	195.57
	(874.73)	(984.74)	(525.52)	(540.33)	(188.33)	(194.20)	(976.60)	(1088.42)
Net crop income (US$/ha)	462.32	509.06	522.48	514.57			68.94	88.75
	(445.53)	(521.47)	(522.90)	(539.68)			(1020.92)	(1120.56)
Household characteristics								
Hh head received pest alert message (1,0)	0.43	0.41	0.62	0.62	0.37	0.35	0.36	0.43
	(0.50)	(0.50)	(0.49)	(0.49)	(0.48)	(0.48)	(0.48)	(0.50)
Male Hh head (1,0)	49.46	49.03	41.37	40.67	44.84	45.19	45.65	45.51
	(12.26)	(12.34)	(11.74)	(11.52)	(13.63)	(13.26)	(14.12)	(14.34)
Age of Hh head (years)	0.84	0.86	0.59	0.58	0.72	0.72	0.45	0.45
	(0.36)	(0.35)	(0.49)	(0.49)	(0.45)	(0.45)	(0.50)	(0.50)
Hh head attended formal education (1,0)	0.17	0.18			0.31	0.31	0.87	0.87
	(0.38)	(0.39)			(0.46)	(0.47)	(0.33)	(0.34)
Hh head owned an ICT-based asset (1,0)	0.96	0.95	0.68	0.69	0.13	0.14	0.80	0.80
	(0.20)	(0.21)	(0.46)	(0.46)	(0.34)	(0.35)	(0.40)	(0.41)
Total farm size (acres)	5.49	5.49	1.50	1.50	2.63	2.76	6.70	6.48
	(3.11)	(3.15)	(1.20)	(1.18)	(2.48)	(2.70)	(12.59)	(11.43)
Experienced weather shocks (1,0)	0.55	0.50	0.37	0.44				
	(0.50)	(0.50)	(0.48)	(0.50)				
Pest/disease outbreaks (1,0)	0.17	0.15	0.63	0.57	0.94	0.91	0.91	0.91
	(0.38)	(0.36)	(0.48)	(0.50)	(0.24)	(0.28)	(0.29)	(0.28)
Observations (no. of households)	880	880	1,413	1,413	819	819	942	942

Note: IPM, integrated pest management. ICT, information and communication technology. Hh, household. Crop income is generated from the three target crops: maize, beans, and tomato. Mean values are shown with standard deviations in parentheses. The exchange rates for a US$ to local currencies at the time of the survey were 6.24, 110, 824 and 10 for Ghana, Kenya, Malawi and Zambia, respectively.

Supplementary Table S4 | Predicted associations between pest alerts and use of IPM practices (Extensive Margins)

Country	Ghana	Kenya	Malawi	Zambia
	(1)	(2)	(3)	(4)
HH received pest alert message (1,0)	0.099***	0.440***	0.189***	0.319***
	(0.036)	(0.023)	(0.067)	(0.035)
Age of HH (log)	0.393**	0.088	0.274	-0.122
	(0.180)	(0.113)	(0.199)	(0.135)
Age of HH square	0.000	0.000	0.000	0.000
	(0.000)	(0.000)	(0.000)	(0.000)
Male HH (1,0)	0.032	0.013	0.048	-0.014
	(0.046)	(0.024)	(0.048)	(0.022)
HH attended formal education (1,0)	-0.018		-0.040	0.121***
	(0.041)		(0.041)	(0.037)
HH owned an ICT-based asset (1,0)	0.069	0.022	0.076	-0.026
	(0.077)	(0.022)	(0.058)	(0.024)
Total farm size (log)	0.058	0.050	0.029	0.027
	(0.049)	(0.036)	(0.036)	(0.042)
Total farm size squared	-0.002**	0.000	0.000	
	(0.001)	(0.002)	(0.000)	
HH experienced weather shocks (1,0)	0.072**	0.124***		
	(0.033)	(0.023)		
	0.190***	0.237***	0.364***	0.076**
HH experienced pest and disease outbreaks (1/0)	(0.031)	(0.025)	(0.092)	(0.029)
Other controls	Yes	Yes	Yes	Yes
District Fixed Effects	No	Yes	Yes	No
F-statistic	6.844	77.719	36.052	84.977
R-squared	0.107	0.298	0.242	0.204
Observations (No. of households)	880	1,408	819	942

Note: Marginal effects estimate from Linear Probability Model (LPM) regression models are shown with robust standard errors clustered at enumeration area in parentheses. Other controls include credit, input subsidy, distance to agro-input dealer or CICs, household size, off-farm work and input expenditure. * $p < 0.1$, ** $p < 0.05$, *** $p < 0.01$.

Supplementary Table S5 | Predicted associations between pest alerts and use of IPM practices (Intensive Margins)

Country	Ghana	Kenya	Malawi	Zambia
	(1)	(2)	(3)	(4)
HH received pest alert message (1,0)	1.081**	1.318***	1.256***	1.246***
	(0.027)	(0.021)	(0.073)	(0.031)
Age of HH (log)	1.143	1.041	1.290*	0.843
	(0.154)	(0.084)	(0.159)	(0.115)
Age of HH square	1.000	1.000	1.000	1.000
	(0.000)	(0.000)	(0.000)	(0.000)
Male HH (1,0)	1.013	1.027	1.058	0.986
	(0.026)	(0.015)	(0.032)	(0.017)
HH attended formal education (1,0)	0.963		0.975	1.100***
	(0.024)		(0.028)	(0.031)
HH owned an ICT-based asset (1,0)	1.086	0.995	1.016	1.001
	(0.046)	(0.014)	(0.061)	(0.014)
Total farm size (log)	1.020	1.057**	0.995	
	(0.029)	(0.022)	(0.030)	
Total farm size squared	0.999	0.999	1.000	
	(0.001)	(0.001)	(0.000)	
	1.057*	1.063***		
HH experienced weather shocks (1,0)	(0.023)	(0.015)		
	1.123***	1.139***	1.288***	1.052*
HH experienced pest and disease outbreaks (1/0)	(0.029)	(0.018)	(0.092)	(0.025)
Other controls	Yes	Yes	Yes	Yes
District Fixed Effects	No	Yes	Yes	No
Wald $\chi2$	160***	622***	574***	440***
Pseudo R-squared	0.015	0.026	0.033	0.011
Observations (No. of households)	880	1,408	819	942

Note: Marginal effects estimate from Poisson regression estimator are shown with robust standard errors clustered at enumeration area in parentheses. To express coefficient estimates as a percentage, we used this formula: (coefficient–1) * 100. For instance, Supplementary Table S5 Column (1) and Row (1) results are interpreted and calculated as (1.081–1) * 100=8.1%. Other controls include credit, input subsidy, distance to agro-input dealer or CICs, household size, off-farm work and input expenditure. * $p < 0.1$, ** $p < 0.05$, *** $p < 0.01$.

Supplementary Table S6 | Predicted associations between pest alerts and maize yield

Country	Ghana	Kenya	Malawi	Zambia
	(1)	(2)	(3)	(4)
HH received pest alert message (1,0)	0.230***	0.197***	0.194**	0.064
	(0.068)	(0.055)	(0.078)	(0.055)
Age of HH (log)	0.513	-0.187	1.063***	0.201
	(0.429)	(0.269)	(0.277)	(0.385)
Age of HH square	0.000	0.000	-0.000***	0.000
	(0.000)	(0.000)	(0.000)	(0.000)
Male HH (1,0)	0.083	0.131***	0.128*	-0.156*
	(0.081)	(0.049)	(0.076)	(0.081)
HH attended formal education (1,0)	0.136*		-0.103	0.012
	(0.082)		(0.074)	(0.096)
HH owned an ICT-based asset (1,0)	-0.087	-0.087*	-0.029	-0.034
	(0.108)	(0.051)	(0.085)	(0.104)
Total farm size (log)	0.102	-0.050	-0.055	
	(0.117)	(0.066)	(0.086)	
Total farm size squared	-0.002	0.004	0.000	
	(0.002)	(0.004)	(0.000)	
HH experienced weather shocks (1,0)	-0.301***	-0.132***		
	(0.082)	(0.042)		
HH experienced pest and disease outbreaks (1/0)	-0.098	-0.033	0.110	0.376**
	(0.105)	(0.060)	(0.099)	(0.148)
Other controls	Yes	Yes	Yes	Yes
District Fixed Effects	No	Yes	Yes	No
F-statistic	4***	6***	21***	11***
R-squared	0.053	0.086	0.233	0.066
Observations (No. of households)	876	885	812	940

Note: Coefficient estimates from ordinary least squares (OLS) and instrumental variable (IV) regression models on the effect of pest alerts (1/0) are shown with robust standard errors clustered at an enumeration area in parentheses. Separate models were estimated for each country. The dependent variables were transformed using an inverse hyperbolic sine (IHS) transformation ($\log(x + (x^2 + 1)^{0.5})$). Other controls include credit, input subsidy, distance to agro-input dealer or CICs, household size, off-farm work and input expenditure* $p < 0.1$, ** $p < 0.05$, *** $p < 0.01$.

Supplementary Table S7 | Predicted associations between pest alerts and bean yield and tomato yield

	Bean yield (IHS)			Tomato yield (IHS)	
Country	Kenya	Malawi	Zambia	Malawi	Zambia
	(1)	(2)	(3)	(4)	(5)
HH received pest alert message (1,0)	0.237***	0.243**	0.091	0.433*	0.329
	(0.084)	(0.115)	(0.142)	(0.220)	(0.384)
Age of HH (log)	1.153***	0.408	-0.194	0.709	-3.177**
	(0.381)	(1.014)	(0.570)	(0.780)	(1.272)
Age of HH square	-0.000***	0.000	0.000	0.000	0.000
	(0.000)	(0.000)	(0.000)	(0.000)	(0.000)
Male HH (1,0)	0.284***	-0.084	-0.196	0.264	0.080
	(0.070)	(0.124)	(0.136)	(0.472)	(0.203)
HH attended formal education (1,0)	0.072	-0.194	0.008	-0.083	0.178
	(0.083)	(0.176)	(0.096)	(0.275)	(0.561)
HH owned an ICT-based asset (1,0)	-0.026	-0.104		0.025	-0.553
	(0.147)	(0.123)		(0.293)	(0.446)
Total farm size (log)	0.004	0.020***		0.737**	
	(0.008)	(0.006)		(0.306)	
Total farm size squared	0.111			-	
	(0.111)			(0.001)	
HH experienced weather shocks (1,0)	-0.156	0.076	0.437		
	(0.106)	(0.142)	(0.267)		
HH experienced pest and disease outbreaks (1/0)	0.237***	0.243**	0.091	0.864**	0.592
	(0.084)	(0.115)	(0.142)	(0.346)	(0.501)
Other controls	Yes	Yes	Yes	Yes	Yes
District Fixed Effects	Yes	Yes	No	Yes	No
F-statistic	6***	6***	5**	.	.
R-squared	0.104	0.104	0.059	0.27	0.212
Observations (No. of households)	405	182	652	90	56

Note: Coefficient estimates from ordinary least squares (OLS) and instrumental variable (IV) regression models on the effect of pest alerts (1/0) are shown with robust standard errors clustered at an enumeration area in parentheses. Separate models were estimated for each country. The dependent variables were transformed using an inverse hyperbolic sine (IHS) transformation ($\log(x + (x^2 + 1)^{0.5})$). Other controls include credit, input subsidy, distance to agro-input dealer or CICs, household size, off-farm work and input expenditure. * $p < 0.1$, ** $p < 0.05$, *** $p < 0.01$.

Supplementary Table S8 | Predicted associations between pest alerts and crop income

Country	Ghana	Kenya	Malawi	Zambia
	(1)	(2)	(3)	(4)
HH received pest alert message (1,0)	0.176*	0.243***	0.259**	0.058
	(0.099)	(0.085)	(0.110)	(0.186)
Age of HH (log)	0.277	1.724***	1.487***	0.167
	(0.466)	(0.413)	(0.269)	(0.982)
Age of HH square	0.000	-0.000***	-0.000***	0.000
	(0.000)	(0.000)	(0.000)	(0.000)
Male HH (1,0)	0.231**	0.073	0.320***	-0.501**
	(0.102)	(0.073)	(0.120)	(0.206)
	0.102		-0.115	0.066
HH attended formal education (1,0)	(0.097)		(0.145)	(0.411)
	0.071	-0.127*	-0.036	0.378**
HH owned an ICT-based asset (1,0)	(0.231)	(0.072)	(0.113)	(0.176)
Total farm size (log)	0.384**	-0.450***	0.215**	
	(0.175)	(0.118)	(0.100)	
Total farm size squared	0.005*	0.012	-0.001**	
	(0.003)	(0.007)	(0.001)	
	-0.494***	-0.255**		
HH experienced weather shocks (1,0)	(0.109)	(0.101)		
HH experienced pest and disease outbreaks	0.031	-0.111	0.047	0.983***
(1/0)	(0.129)	(0.101)	(0.122)	(0.327)
Other controls	Yes	Yes	Yes	Yes
District Fixed Effects	No	Yes	Yes	No
F-statistic	21***	3***	11***	20***
R-squared	0.179	0.054	0.187	0.137
Observations (No. of households)	880	942	819	942

Note: Coefficient estimates from ordinary least squares (OLS) regression models on the effect of pest alerts (1/0) are shown with robust standard errors clustered at an enumeration area in parentheses. Separate models were estimated for each country. The dependent variables were transformed using an inverse hyperbolic sine (IHS) transformation ($\log(x + (x^2 + 1)^{0.5})$). Other controls include credit, input subsidy, distance to agro-input dealer or CICs, household size, off-farm work and input expenditure. *, **, *** indicate statistical significance at 10%, 5%, and 1% level, respectively.

Supplementary Table S9 | Predicted associations between pest alerts and net crop income

Dependent variable	Net crop income (IHS)
Estimator	OLS
	(1)
Ghana (n = 880)	0.253***
	(0.080)
Kenya (n = 1,408)	-0.420*
	(0.238)
Malawi (n = 819)	
Zambia (n = 949)	-0.118
	(0.439)

Note: Coefficient estimates from ordinary least squares (OLS) regression models on the effect of pest alerts (1/0) are shown with robust standard errors clustered at an enumeration area in parentheses. Due to data limitation on major input expenditure in Malawi, we could not calculate net crop income. Separate models were estimated for each country. The dependent variables were transformed using an inverse hyperbolic sine (IHS) transformation ($\log(x + (x^2 + 1)^{0.5})$). For brevity, we do not present full model results with relevant confounding factors in this Supplementary Table S9. * $p < 0.1$, ** $p < 0.05$, *** $p < 0.01$.

Supplementary Table S10 | Predicted associations between pest alerts and use of IPM practices: Summarized Probit Models

Estimator	Extensive margins
	Probit
	(1)
Ghana (n = 880)	0.331***
	(0.116)
Kenya (n = 1,408)	1.340***
	(0.078)
Malawi (n = 770)	0.588***
	(0.201)
Zambia (n = 942)	1.448***
	(0.193)

Note: The dependent variable in all the models is either a binary (1/0) variable or a count variable for integrated pest management (IPM) practices. Marginal effects estimate from probit regression models are shown with robust standard errors clustered at enumeration area. *** $p < 0.01$.

Supplementary Table S11 | Predicted associations between pest alerts and use of IPM practices: Summarized Weighted Models

Estimator	Extensive margins	Intensive margins
	LPM	Poisson
	(1)	(2)
Ghana (n = 880)	0.084**	1.070**
	(0.038)	(0.026)
Kenya (n = 1,408)	0.426***	1.299***
	(0.030)	(0.026)
Malawi (n = 819)	0.163**	1.219**
	(0.082)	(0.082)
Zambia (n = 942)	0.303***	1.243***
	(0.039)	(0.036)

Note: The dependent variable in all the models is either a binary (1/0) variable or a count variable for integrated pest management (IPM) practices. Marginal effects estimate from Linear Probability Model (LPM) and Poisson estimators are shown with robust standard errors clustered at enumeration area. To express coefficient estimates as a percentage in columns (2) of Supplementary Table S11, we used this formula: (coefficient–1) × 100. * $p < 0.1$, ** $p < 0.05$, *** $p < 0.01$.

Supplementary Table S12 | Predicted associations between pest alerts and crop productivity and income: Summarized Weighted Regression Model Results

	Maize yield (IHS)	Bean yield (IHS)	Crop income (IHS)
	(1)	(2)	(3)
Ghana (n = 880)	0.194**		0.158
	(0.075)		(0.117)
Kenya (n = 942)	0.202***	0.242***	0.294***
	(0.054)	(0.087)	(0.090)
Malawi (n = 819)	0.266***	0.188	0.311**
	(0.092)	(0.131)	(0.127)
Zambia (n = 942)	0.078	0.180	0.143
	(0.054)	(0.139)	(0.166)

Note: Coefficient estimates from ordinary least squares (OLS) regression models on the effect of pest alerts (1/0) are shown with robust standard errors clustered at an enumeration area in parentheses. Due to data limitation on major input expenditure in Malawi, we could not calculate net crop income. Separate models were estimated for each country and for each of the three dependent variables. The dependent variables were transformed using an inverse hyperbolic sine (IHS) transformation ($\log(x + (x^2 + 1)^{0.5})$). For brevity, we do not present full model results with relevant confounding factors in this Supplementary Table S12. * $p < 0.1$, ** $p < 0.05$, *** $p < 0.01$.

Supplementary Table S13 | Predicted associations between pest alerts and use of IPM practices, maize yield, and income: IPWRA models

	Use of IPM practices (1/0)	Maize yield (IHS)	Crop income (IHS)
	(1)	(2)	(3)
Ghana (n = 880)	0.090***	0.223***	0.141
	(0.032)	(0.065)	(0.086)
Kenya (n = 1409)	0.415***	0.188***	0.259***
	(0.025)	(0.056)	(0.079)
Malawi (n = 819)	0.212***	0.056	0.074
	(0.036)	(0.071)	(0.098)
Zambia (n = 942)	0.321***	0.074	0.087
	(0.028)	(0.068)	(0.158)

Note: ATE, average treatment effect. IPWRA, Inverse probability weighted regression adjustment. For brevity, we do not present full model results with relevant confounding factors in this Supplementary Table S13. * $p < 0.1$, ** $p < 0.05$, *** $p < 0.01$.
